# Advances in Translational Research and Clinical Care in Pancreatic Cancer: Where Are We Headed?

**DOI:** 10.1155/2019/7690528

**Published:** 2019-02-04

**Authors:** Vikas Satyananda, Rohan Gupta, Danielle M. Hari, James Yeh, Kathryn T. Chen

**Affiliations:** ^1^Division of Surgical Oncology, Department of Surgery, Harbor-UCLA Medical Center, USA; ^2^Division of Medical Oncology, Department of Medicine, Harbor-UCLA Medical Center, USA

## Abstract

While significant advances have been made in the treatment of many different solid tumors, pancreatic cancer remains a glaring exception. Overall 5-year survival rates for pancreatic cancer remain in the single digits. While newer chemotherapy regimens such as FOLFIRINOX and nab-paclitaxel/gemcitabine have demonstrated modest improvement in survival benefit for metastatic disease and have improved the resectability rates of previously borderline or locally advanced tumors, clinically significant improvements from immunotherapy and targeted therapy remain to be demonstrated. Regardless, a wealth of basic science research in pancreatic cancer has been directed at understanding its aggressive biology and its resistance to therapy. We present a brief summary of key areas of laboratory research and its translation to clinical care.

## 1. Introduction

In the past two decades, there have been significant advances in the treatment of different cancer types, particularly with the exploding field of targeted therapy and immunotherapy. From the success story of imatinib in chronic myelogenous leukemia to programmed cell death protein (PD-1) inhibition in melanoma to chimeric antigen receptor T cell (CAR-T) therapy in refractory lymphoma, patients who were refractory to conventional cytotoxic agents now have treatment options that are effective and durable. On the other hand, advances in the treatment of pancreatic cancer have been frustratingly slow. Pancreatic cancer is notoriously aggressive and rarely curable, and these factors in turn curb research efforts. Pancreatic tumors are immune-quiescent, and single-agent immunotherapies have failed to show a significant clinical response [1–4]. This is due in part to a tumor microenvironment, characterized by a dense desmoplastic stroma, which demonstrates high inflammatory cell expression and limits intratumoral infiltration with effector T cells [5–8]. Notable efforts have been made to understand how we can break down this stromal barrier and stimulate immune response to pancreatic tumors.

While immunotherapy is at the forefront of translational research efforts, other key areas of interest include targeted therapies against tumor cells and the extracellular matrix, pathogenesis of pancreatic cancer, and methods of early detection. In this paper, we outline the trends in translational research in pancreatic cancer with respect to these elements.

## 2. Pathogenesis

The development of pancreatic cancer is thought to be multifactorial, with several recognized risk factors, including smoking, alcohol, diabetes, pancreatitis, and, most significantly, family history [8–10]. While hereditary gene mutations may contribute up to 10% of pancreatic cancers, the majority of gene alterations are somatic. Multiple genes have been identified which affect the molecular pathogenesis of pancreatic cancer, although with some heterogeneity. The tumor suppressor genes SMAD4 and TP53 and the protooncogene KRAS are commonly mutated and lead to progression from benign pancreatic intraepithelial neoplasia to infiltrative tumor [11–13]. Unfortunately, the identification of individual genetic alterations has not been particularly useful in therapeutic targeting, and clinical applications remain limited [4, 14, 15]. With whole genomic sequencing, molecular subtypes of pancreatic cancer are now better defined [13, 16–18]. One study described an average of 48 somatic gene mutations in pancreatic cancer—considerably less than breast, colorectal, or lung cancers [13]. As found in other whole genome cancer studies, this is consistent with the observation that normal pancreatic cells divide infrequently and are likely subject to fewer mutagenic processes (e.g., tobacco in lung cancer) [19]. One study identified 12 core signaling pathways as genetically targeted in over two-thirds of the 24 tumors sequenced, providing a framework for the molecular pathogenesis of pancreatic cancer [13]. Other genomic analyses have identified distinct molecular subtypes within pancreatic cancer, highlighting different pathways in the evolution of these tumors [18, 20]. Known precursors to pancreatic cancer, such as pancreatic intraepithelial neoplasia (Pan-IN) and intraductal mucinous papillary neoplasm (IPMN), virtually all harbor gene mutations [21, 22]. These findings may help direct biomarker detection for diagnosis for those precursor lesions that may progress to invasive adenocarcinoma.

In terms of germline mutations, four genes have been known to cause familial pancreatic cancer: BRCA, p16/CDKN2A, STK11, and PRSSI [16]. However, new and different germline mutations, including PALB2 and ATM [23, 24], have been recently identified. These discoveries allow for the appropriate counseling of patients who are at risk for other cancers and may also provide a mechanism for screening for pancreatic cancer, although this role is not yet well defined.

## 3. Early Detection

About 80-85% of patients with pancreatic adenocarcinoma are diagnosed with locally advanced or metastatic disease. Only 15-20% of patients are found to have resectable disease, with radical surgical resection improving 5-year survival from 5% to 20-25% [25]. Hence, early detection of pancreatic cancer is vital. Because of the relatively low incidence of pancreatic cancer, screening of pancreatic cancer is unlikely to be feasible in the general population. Certain circumstances may benefit from screening, including patients with a familial history, genetic predisposition syndromes associated with pancreatic cancer, patients with incidentally discovered indeterminate pancreatic cysts, or surveillance following resection of an IPMN, considered to be a field defect within the organ.

An ideal method of detection would employ a serum biomarker panel, which would be relatively noninvasive and cost-effective. Traditional serum tumor markers include serum CA19-9 and CEA. CA 19-9 is the most widely used biomarker of PDAC today. However, CA 19-9 is elevated in only 65% of patients with resectable PDAC and it can also be elevated in many other conditions, both benign (pancreatitis, cirrhosis, and obstructive jaundice from benign etiologies) and malignant (colorectal, gastric, and uterine cancers) [26]. As such, CA19-9 levels are currently most useful in assessing response to chemotherapy or detecting recurrence in patients who had elevated pretreatment levels.

Advances in proteomics with liquid chromatography-tandem mass spectrometry have allowed the identification of many potential biomarkers in the plasma of patients via high-throughput quantification. Several candidate protein molecules, including C4b-binding protein alpha chain (C4BPA), help in distinguishing chronic pancreatitis from PDAC and biliary tract cancers. Insulin-like growth factor-binding protein (IGFBP) 2 and IGFBP 3 in combination with CA 19-9 were predictive of PDAC in a sample of over 200 patients [27]. Several other studies with promising biomarkers to aid in differentiating between benign and malignant lesions require validation on a larger scale.

Another innovative method of investigating potential protein-based biomarker panels includes two independent studies that performed meta-analyses of pancreatic cancer transcriptome studies to identify multigene classifiers or pancreatic cancer [28, 29]. These studies both further validated their multigene classifiers against mouse models, formalin-fixed paraffin-embedded tissue samples, and pancreatic cancer tissue microarrays. Between the two studies, there were four genes which overlapped (AHNAK2, SERPINB5, TMPRSS4, and POSTN) [28, 29], which were able to differentiate pancreatic cancer from benign pancreatic conditions, as well as provide prognostic information. Translation to real-time clinical scenarios remains to be seen.

Metabolomics involves the comprehensive study of metabolites in biological specimens. Various metabolites have been measured in conjunction with CA 19-9, including glucitol, palmitate, xylitol, inositol, histidine, sphingagine-1 phosphate, sphingomyelin d17:1, and pyruvate, and have shown increased diagnostic accuracy of PDAC detection when compared with CA 19-9 alone [30].

Noncoding RNAs (ncRNAs), both small ncRNAs (sncRNAs, <200 bases) and long ncRNAs (lncRNA, > 200 bases), are also interesting biomarker candidates. ncRNAs regulate gene expression at a posttranslational level and may be representative of epigenetic alterations that drive tumorigenesis. MicroRNAs (miRNAs) are a group of sncRNAs, which have shown potential as markers for early detection of PDAC; they can be isolated from serum, plasma, pancreatic juice, stool, urine, and saliva [31]. Several studies have shown miRNA or their panels in plasma or serum have potential diagnostic value (miR-1290, miR-486-5p) with more sensitivity than CA19-9 [32, 33].

Circulating tumor cells (CTCs) have been long investigated as potential biomarkers. CTCs are cells derived from a primary cancer that have entered the vasculature and circulate within the bloodstream to seed distant organs. CTCs are currently used as a prognostic biomarker in metastatic breast, prostate, and colorectal cancers; however, they have not yet been established as a method for screening or diagnosis [34]. In pancreatic cancer, the sensitivity of CTCs in the detection of nonmetastatic pancreatic cancer ranges from 5 to 75% in studies with mostly small sample sizes and is not currently employed for diagnosis [35–37].

There are many other biomarkers that are under investigation, including circulating free DNA, cytokines, and exosomes (Table 1). In addition to its clinical utility for early detection, these biomarkers may be important in understanding the pathogenesis of pancreatic cancer and may provide insights towards therapeutic targets.

## 4. Targeted Therapies

Understanding the molecular pathogenesis of pancreatic cancer allows for identification of multiple areas in cell signaling or tumor formation to target therapies, as well as to identify molecular signatures that may respond to current cytotoxic regimens. Prior attempts at targeted therapy have been largely unsuccessful, including various points of the KRAS signaling cascade. A phase II study evaluating the use of cetuximab, an antibody which binds the epidermal growth factor receptor (EGFR) expressed on tumor cells, found no improvement in survival [38], despite prior apparent antitumoral effects in mice [39, 40]. Aside from transmembrane receptor proteins, other therapies that have been unsuccessful in clinical trials include targeted agents against the Notch and JAK/STAT signaling pathways [41].

There has been some progress with targeting BRCA mutations, present in 5-10% of pancreatic cancers. Alterations in BRCA, a tumor suppressor gene, have been targeted with poly ADP ribose polymerase (PARP) inhibitors. Although several case studies reported responses to PARP inhibition in patients with BRCA mutations, a phase II study evaluating veliparib as monotherapy in patients previously treated with cytotoxic therapy showed no confirmed response [42–44]. There are ongoing trials evaluating combination therapy with chemotherapy and PARP inhibition [45, 46], which may yield more promising results. Hyaluronic acid is a hydrophilic glycosaminoglycan whose production within the tumor leads to increased interstitial tumor pressure and thus limits the access of potentially effective circulating anticancer drugs due to reduced tumor perfusion [47]. Pegvorhyaluronidase alfa (PEGPH20) targets tumor microenvironment by degrading excessive hyaluronan in the tumor microenvironment and leads to improved delivery of anticancer therapy to the tumor cells. The phase II HALO 202 randomized 279 patients with previously untreated metastatic pancreatic ductal adenocarcinoma to PEGPH20 plus nab-paclitaxel/gemcitabine or nab-paclitaxel/gemcitabine. In patients with HA-high tumors (34%), there was improvement in progression-free survival (9.2 months vs. 5.2 months) and overall survival 11.5 months vs. 8.5 months in PEGPH20 arm. There were increased muscle spasms (13% vs. 1%), neutropenia (29% vs. 18%), and myalgia (5% vs. 0%) in PEGPH20 arm [48]. The phase III trial HALO-109-301 is currently ongoing [49]. In contrast, an early phase trial of PEGPH20 with mFOLFIRINOX was closed to accrual after a futility analysis showed a hazard ratio for OS of 0.44 in favor of mFOLFIRINOX [50]. The investigators recommended not studying this drug further with FOLFIRINOX. An early phase trial evaluating the pharmacodynamics, safety, and efficacy of PEGPH20 in combination with avelumab in adult patients with chemotherapy-resistant advanced or locally advanced pancreatic ductal adenocarcinoma is ongoing [51].

Napabucasin (NAPA) is an inhibitor of cancer stemness and STAT3 pathways, which lead to cancer stem cell viability. An early phase study, presented at the 2018 ASCO meeting, showed that oral 240 mg bid NAPA in combination with nab-paclitaxel and gemcitabine was well tolerated with disease control rate which was observed in 46 out of 59 patients. There were two complete responses and 26 partial responses [52]. This combination is now being further investigated in an ongoing phase III study [53].

## 5. Immunotherapy

Monotherapy with T cell-directed immunotherapy via checkpoint inhibitors has been largely unsuccessful. Agents that have been clinically tested include ipilimumab and tremelimumab (anti-CTLA-4) and BMS-936559 (anti-PD-L1); these agents all failed to show benefit against pancreatic cancer in terms of overall survival [2, 3, 54]. More recently, monotherapy with pembrolizumab (anti-PD-1) has been shown to be successful in the treatment of a range of cancers with mismatch repair (MMR) deficiency, due to high expression of mutant neoantigens that render these tumors responsive to checkpoint blockade [55, 56]. In one study, of eight patients with MMR-deficient pancreatic cancer, 25% of patients had a complete response and 37% percent had a partial response [56]. However, less than 4% of all pancreatic cancers are shown to be MMR-deficient, compared to nearly 10% for gastric cancer and 6% for colorectal cancer [56].

Sherman et al. showed that the vitamin D receptor is expressed in stroma from human pancreatic tumors. Treatment with a vitamin D agonist reduced markers of inflammation and fibrosis in pancreatitis and human tumor stroma and acted as a sensitizing agent to PD1 blockage agents in treatment of pancreatic cancer [57]. A clinical trial evaluating the role of maintenance immunotherapy and paricalcitol after best response to cytotoxic chemotherapy is ongoing [58].

Vaccine therapies are also under active investigation and have been shown to induce T cell responsiveness against tumor cells. Algenpantucel-L, created from allogenic irradiated pancreatic cancer cells to express *α*-GT to cause hyperacute rejection, demonstrated efficacy in animal models of melanoma and subsequently in a phase II trial in pancreatic cancer [59]. Unfortunately, a phase III trial designed to evaluate the effectiveness of algenpantucel-L in combination with a cytotoxic regimen failed to show an improvement in overall survival (press release, NewLink Genetics). GVAX-CSF, a vaccine that expresses GM-CSF and induces an immune response against the tumor, inhibited of tumor growth in animal and cell models, but failed to demonstrate efficacy in a phase II trial compared to single-agent chemotherapy [60].

Strategies are being developed to unlock the potential of immunotherapy with combination therapy, particularly in MMR-proficient pancreatic cancers. Clinical trials evaluating T cell expansion with vaccine therapy followed by PD-1/PD-L1 inhibition are ongoing [61, 62]. Other trials are evaluating the efficacy of induction or combination chemotherapy to activate T cells, alongside checkpoint inhibition, as well as radiation therapy or targeted therapies alongside checkpoint inhibition (Table 2). While these combinations or strategies have shown efficacy in animal models, their benefit from clinical trials remains to be seen [63, 64]. In the interim, the search for novel immune checkpoint molecules continues.

## 6. Conclusion

While many advances have been made in understanding pancreatic cancer in terms of gene expression, tumor microenvironment, and molecular pathogenesis, the translation from bench to clinical management remains a challenge. Although research continues to uncover new pathways to investigate and target for therapy, very few of these targets have borne out in clinical trials thus far. A biomarker panel to reliably differentiate pancreatic precursor lesions that will progress to malignancy from those that are benign has also remained elusive. The most promising area in treating pancreatic cancer appears to be stimulating the immune response to pancreatic cancer by breaking through the tumor microenvironment and enhancing directed antitumor T cell responses with combination therapies. The results of these ongoing efforts will be revealed in the near future.

## Figures and Tables

**Table 1:** Selected proposed biomarkers.

	Selected biomarkers of interest	Studied in	Ref.
Antigens	CA19-9, CEA	Plasma	Locker et al. [26]
Proteins	IGFBP 2, IGFBP 3	Plasma	Yoneyama et al. [27]
	SYCN, REG1B, AGR2	Plasma	Makawita et al. [65]
	ICAM1, OPG	Plasma	Brand et al.
	MMP-9, DJ-1, A1BG,	Pancreatic fluid	Tian et al. [66]
	thrombospondin-2	Plasma	Kim et al.
Metabolites	Glucitol, palmitate, xylitol	Plasma	Mayerle et al. [30]
ncRNAs	miR-1290	Plasma	Cao et al. [32]
	miR-486-5p	Plasma	Li et al. [33]
CTCs		Plasma	Bidard et al. [35], de Albuquerque et al. [36], Kulemann et al. [37]
cfDNA		Plasma	Tjensvoll et al. [67]
Exosomes	GPC1+crExos	Plasma	Melo et al. [68]

ncRNAs: noncoding RNAs; CTC: circulating tumor cells; cfDNA: circulating free DNA.

**Table 2:** Selected active combination immunotherapy trials.

	Pancreatic cancer classification	Clinical trial no.
Vaccine+IO		
GVAX+nivolumab	Surgically resectable	NCT02451982
Vaccine+IO+radiation		
GVAX+pembrolizumab+SBRT	Locally advanced	NCT02648282
GVAX+nivolumab+SBRT	Borderline resectable	NCT03161379
IO+chemotherapy		
Atezolizumab+chemotherapy	Metastatic	NCT03193190
IO+radiation		
Nivolumab+radiation	MSS pancreatic cancer	NCT03104439
IO+chemoradiation		
Pembrolizumab+chemoradiation	Borderline resectable	NCT02305186
Nivolumab+chemo+SBRT	Locally advanced	NCT03563248

IO: immunotherapy; SBRT: stereotactic body radiation therapy; MSS: microsatellite stable.
